# Extrinsic extracellular DNA leads to biofilm formation and colocalizes with matrix polysaccharides in the human pathogenic fungus *Aspergillus fumigatus*

**DOI:** 10.3389/fmicb.2013.00141

**Published:** 2013-06-06

**Authors:** Iordana Shopova, Sandra Bruns, Andreas Thywissen, Olaf Kniemeyer, Axel A. Brakhage, Falk Hillmann

**Affiliations:** ^1^Department of Molecular and Applied Microbiology, Leibniz Institute for Natural Product Research and Infection Biology – Hans Knöll InstituteJena, Germany; ^2^Department of Microbiology and Molecular Biology, Institute of Microbiology, Friedrich Schiller UniversityJena, Germany; ^3^Integrated Research and Treatment Center, Center for Sepsis Control and Care, Jena University HospitalJena, Germany

**Keywords:** aspergillus, cystic fibrosis, extracellular DNA, extracellular matrix, fungal biofilm

## Abstract

The environmentally acquired fungal pathogen *Aspergillus fumigatus* causes a variety of severe diseases. Furthermore, it is often found colonizing the respiratory tract of patients suffering from cystic fibrosis. Conidia of this filamentous fungus adhere to substrate surfaces and germinate to form biofilms comprised of dense hyphal networks embedded in an adhesive extracellular matrix (ECM), built predominantly of polysaccharides. These fungal microconsortia are likely to be of clinical relevance, as they have also been observed during growth in the host and they confer drastically reduced susceptibility to antifungals. Little is known about environmental factors or signals contributing to the formation and structural organization of this polysaccharide matrix. Extracellular DNA (eDNA) is an abundant molecule in the mucus-rich surfaces in the lungs of cystic fibrosis patients. Here, we studied its influence on the biofilm establishment and progression of *A. fumigatus*. Using an *in vitro* biofilm model eDNA was identified as an efficient biofilm inducer promoting conidial surface adhesion and polysaccharide ECM production. Confocal laser scanning microscopy revealed entirely different ECM architectures depending on the substrates used for biofilm induction. In the presence of serum, adhesive polysaccharides were mainly localized to the hyphal tips appearing as cohesive threads or “halo” areas agglutinating the hyphae. Exogenous DNA altered the structural organization of the biofilm specifically by colocalizing to a grid-like bottom layer of ECM. These results indicate that biofilm formation in *A. fumigatus* is shaped by certain substrates and in response to host environmental signals.

## Introduction

Only a few species of the wide spread filamentous fungi are opportunistic pathogens causing often life-threatening diseases in higher animals and humans. *Aspergillus fumigatus* can be regarded as a model organism of this group, causing primarily pulmonary infections like aspergilloma, cystic fibrosis (CF) associated allergic bronchopulmonary aspergillosis (ABPA) or even often fatal invasive aspergillosis in immunocompromised individuals (Latgé, 2001; Brakhage, 2005; Nivoix et al., 2008). The apparent lack of specific virulence factors has also contributed to the fact that increasing attention is drawn to the infection process and the general life style of this saprophytic mold within the human host (Tekaia and Latgé, 2005). Lung infection propagation is due to conidia of which several hundred are usually inhaled every day. When reaching preformed lung cavities, or the mucus rich surfaces of lung epithelia from CF patients, inhaled conidia can escape the innate immune responses and germinate to form hyphae. Under all growth conditions analyzed so far, hyphae revealed complex multicellular communities characterized by a striking resemblance to biofilms formed by *Candida albicans* (Beauvais et al., 2007; Mowat et al., 2009; Loussert et al., 2010). *A. fumigatus* fungal microconsortia were also observed during surface adherent growth *in vitro*, conforming to essentially all requirements defined for bacterial biofilms (Mowat et al., 2009; Ramage et al., 2009). Overall, conidia as well as hyphae of *A. fumigatus* have adhesive and cohesive properties, which are at least in part mediated by an extensive polysaccharide based extracellular matrix (ECM). This ECM covers standard aerially grown colonies but is also present *in vivo* during host colonization, embedding a highly structured hyphal network, characterized by an increase in resistance to the most prominent antifungal drugs (Beauvais et al., 2007; Mowat et al., 2007; Loussert et al., 2010). In agreement with these data, similar results on ECM production, hyphal organization and resistance to antifungals were also obtained for an extended model demonstrating that *A. fumigatus* biofilms are formed on the surface of human bronchial epithelial and CF bronchial epithelial cells (Seidler et al., 2008).

*A. fumigatus* biofilms have been subject to several “omics”-based studies toward a comprehensive overview of the gene expression on a global scale (Muszkieta et al., 2013). In a first approach to understand the molecular basis of biofilm formation in this fungus, we have recently compared global gene and protein expression in planktonic- and biofilm-growing hyphae (Bruns et al., 2010b). A high number of genes involved in primary energy metabolism were found to be highly expressed during early stages of the biofilm covering the first 24 h. Matured biofilms had reduced metabolic activity, but in turn increased production of secondary metabolites. This was most pronounced for gliotoxin-a mycotoxin, whose exact role in virulence is yet to be defined. Recent data suggest the immunosuppressive properties of gliotoxin by targeting downstream molecular pathways involving NF-κB in neutrophils and other phagocytes (Spikes et al., 2008; Scharf et al., 2012). A high-level-expression of secondary metabolite gene clusters was also observed during colony growth of the fungus, revealing striking similarities to growth in biofilms (Gibbons et al., 2012).

The importance of extracellular DNA (eDNA) in biofilms of Gram-positive and Gram-negative bacterial pathogens is widely recognized, but first evidence for its role in inducing and shaping biofilms was given for the opportunistic pathogen *Pseudomonas aeruginosa*. Here, eDNA triggered the initial establishment of the biofilm, whose structural integrity remained sensitive to DNase I treatment (Whitchurch et al., 2002). The DNA incorporated into the ECM was found to be of chromosomal origin following quorum-sensing-controlled lysis of a bacterial subpopulation (Allesen-Holm et al., 2006). More recent results for *P. aeruginosa* also indicated that eDNA chelates cations resulting in a cellular stress response leading to a drastic increase in antibiotic resistance (Mulcahy et al., 2008). The limited knowledge regarding the function and localization of eDNA during fungal biofilm development originates almost exclusively from results obtained for a few *Candida sp*. In *C. albicans*, DNA was not found to be a major constituent of the biofilm. However, treatment with DNase I resulted in the detachment of cells and in an adverse situation, addition of exogenous DNA to mature biofilms led to a significant increase in biomass (Al-Fattani and Douglas, 2006; Martins et al., 2010). Only very recently, it was shown that also in *A. fumigatus* DNA is released mainly during later stages in biofilm development, conferring a functional role in stability (Rajendran et al., 2013). This DNA release was mediated by autolysis following an increased activity of chitinases and resulted in reduced stress tolerance and lower susceptibility to antifungals. Biomass production in biofilms was also enhanced by DNA from exogenous sources.

Interestingly, free, host-derived DNA is of particular clinical significance contributing to the high viscosity of the mucus in the lungs of CF patients, which is partially counteracted by the administration of nebulized human recombinant DNase I (Shak et al., 1990; Cantin, 1998). CF-sputum DNA mainly originates from necrotic tissues and the release of decondensed chromatin from invading neutrophils undergoing NETosis (Lethem et al., 1990; Papayannopoulos et al., 2011). The formation of neutrophil extracellular traps (NETs) is a part of a suicidal cellular program designed to locally restrict or even kill pathogenic bacteria (Brinkmann et al., 2004; Fuchs et al., 2007). However, NET structures are not only induced by prokaryotes, but were also observed in several *in vivo* models during pulmonary infection with the most prevalent fungal pathogens *C. albicans* and *A. fumigatus* (Urban et al., 2009; Bruns et al., 2010a). In contrast to other pathogens, *A. fumigatus* did not show significantly reduced viability during NETosis (Bruns et al., 2010a). Hence, this study was designed to provide insights into how germination and growth of *A. fumigatus* are affected by the presence of exogenous DNA giving first evidence that it promotes surface adhesion of conidia, biofilm formation and colocalizes with the ECM.

## Materials and methods

### Organisms and culture conditions

All biofilm assays were carried out with conidia harvested from aerial static cultures of *A. fumigatus* CEA17 Δ*akuB*^Ku80^ (Da Silva Ferreira et al., 2006), a derivative of the sequenced clinical isolate CEA10 (d'Enfert, 1996). Conidia were harvested from *Aspergillus* Minimal Medium (AMM) agar plates as previously described (Weidner et al., 1998). Spore suspensions were either freshly harvested or stored for no longer than 1 week at 4°C to set up biofilms or to inoculate liquid cultures with RPMI or AMM. *Escherichia coli* DH5α carrying the high-copy pUC18 plasmid was grown in 500 ml of LB medium with 100 μg/ml of ampicillin and served as a source of plasmid DNA.

### Biofilm growth of *A. fumigatus*

Biofilms of *A. fumigatus* were set up as described previously by Mowat et al. (2007) and Seidler et al. (2008) with minor modifications. Briefly, spore suspensions were diluted to densities of 1 × 10^3^−1 × 10^6^ spores in total per well in HEPES-buffered RPMI 1640 with L-glutamine (PAA, Pasching, Austria) and dispensed on flat, presterilized, polystyrene surfaces of 6-well plates or Petri dishes (VWR International bvba, Leuven, Belgium). Several media were tested, e.g., RPMI, RPMI with 10% (vol/vol) heat inactivated FCS (PAA, Pasching, Austria) and RPMI with DNA from various sources (see below). FCS was previously shown to provide sufficient nutrients to promote growth of a hyphal network and induce biofilm formation (Seidler et al., 2008). Following an initial adherence phase of 4 h during static incubation in RPMI at 37°C, unbound conidia were washed three times with 10 ml sterile PBS-Tween 80 solution (Merck, Darmstadt, Germany). Fresh RPMI medium with the indicated additives was added to the adhered conidia and static submerged cultures were grown up to 48 h at 37°C.

### DNA in biofilm assays

If not stated otherwise, commercially available DNA from herring sperm (Sigma-Aldrich, Munich, Germany) was added to RPMI without further purification. This sheared DNA was readily available in larger amounts and is comprised of largely crude oligonucleotides of less than 50 bp in length, as stated in the manufacturer's guidelines. Purity and size were verified by agarose gel electrophoresis as well as spectrophotometric analysis. Other sources of DNA included fungal genomic DNA and plasmid DNA. Fungal genomic DNA was isolated from 200 ml of overnight cultures of *A. fumigatus* grown in AMM with 1% (w/v) of glucose as a carbon source, using standard methods according to Sambrook and Russell (2001). Where indicated, crude DNA preparations were subjected to two successive rounds of extraction with equal volumes of phenol/chloroform/isoamyl alcohol (Carl Roth, Karlsruhe, Germany) to remove contaminations of DNA binding proteins such as histones. These DNA preparations contained larger fragments of DNA in the range of 10–30 kbp. Plasmid DNA was isolated from overnight cultures of *E. coli* DH5α originally transformed with the high-copy pUC18 vector. Large-scale plasmid purification was carried out using the GeneJET plasmid maxiprep Kit (Thermo Fisher Scientific, Dreieich, Germany) according to the manufacturer's instructions. The concentration of DNA added during biofilm development was 1 mg/ml and this selection was based on previous data obtained for concentrations determined in sputum or BAL fluid of CF patients reaching up to 9.5 mg/ml (Brandt et al., 1995). Enzymatic digestion of DNA was achieved by treatments with DNase I (Sigma Aldrich, Munich, Germany), which was added at indicated time points of biofilm development to RPMI up to a final concentration of 0.2 mg/ml.

### Biomass production of biofilms

As a measure of fungal biomass production the dry weight of hyphae produced under static and “planktonic” conditions was determined. *A. fumigatus* was either cultivated in shaken liquid cultures or grown as a biofilm from adherent spores on polystyrene Petri dishes with different media. At indicated time points, mycelia were harvested by scraping from the surface of the Petri dishes or filtration through Miracloth (Merck, Darmstadt, Germany). Both preparations were washed three times with H_2_O and dried up to a constant weight.

### Adhesion test

Direct determination of colony forming units (CFUs) is difficult due to the cohesive properties of germinating conidia (Fontaine et al., 2010). Therefore, the conidial adhesion to polystyrene surfaces was quantified directly by visual counting using inverse light microscopy. Conidia were diluted to 2 × 10^6^ spores/well in RPMI alone or with additives and allowed to adhere to the bottom of 6-well plates. At the end of 4 h of adherence phase, medium was aspirated removing the unbound spores, followed by three sequential washing steps with 10 ml sterile PBS-Tween 80 solution (Merck, Darmstadt, Germany) to remove any remaining unbound spores. For all conditions the number of surface bound conidia in at least 4 fields of defined surface dimension was determined and is expressed as the percentage of total spores in a single assay.

### Quantification of extracellular matrix

Polysaccharides of the ECM in fungal biofilms can be readily visualized by the lectin conjugate Concavalin A Alexa-fluor 488 (CAAF, Life Technologies, Darmstadt, Germany) allowing ECM measurements in a semi-quantitative fluorescent microtiter plate assay. CAAF stains primarily α-mannopyranosyl and α-glucopyranosyl residues in polysaccharides present in the ECM and the fungal cell wall. It was previously employed to stain the ECM of fungal biofilms in CLSM, and also for the quantitative analysis of biofilms (Yang et al., 2006; Chandra et al., 2008; Seidler et al., 2008). For this purpose, biofilms of *A. fumigatus* were cultivated on black 96-well plates (Brand, Wertheim, Germany) using standardized fresh spore suspensions in 200 μl at final concentrations of 10^4^ and 10^5^ spores per well. Biofilms were grown in static conditions for 48 h at 37°C. Fluorescent labelling of the samples was achieved by adding 100 μl of 25 μg/ml CAAF in PBS followed by 45 min incubation at 37°C on a plate shaker at 250 rpm. Biofilms were then carefully washed three times with sterile PBS to preserve the biofilm architecture and fluorescence intensity was measured on a FluoStar Optima plate reader (BMG LabTechnologies, Ortenberg, Germany) using an excitation filter of 485 nm and an emission filter of 520 nm. Stock solutions of 5 mg/ml CAAF were stored at -20°C and thawed immediately prior use.

### Biofilm viability testing

The overall viability of fungal biofilms was monitored as an increase in fungal respiration. Similar to the widely used XTT assay (Mowat et al., 2007; Pierce et al., 2008; Seidler et al., 2008), resazurin undergoes a color change upon its metabolic reduction from blue to the pink resorufin and has successfully been used as a respiration indicator in bacterial biofilms (Mariscal et al., 2009). The resazurin assay can be employed either as a fluorimetric or a colorimetric technique. We measured the decrease in absorbance of resazurin at a wavelength of 600 nm. Biofilms were set up in HEPES-buffered RPMI 1640 with L-glutamine (PAA, Cölbe, Germany), lacking the pH indicator phenol-red, at densities of 10^4^ and 10^5^ spores per well in flat-bottomed 24-well NunclonΔ Multidishes (Thermo Fisher Scientific, Dreieich, Germany). After 4 h adhesion phase and washing with PBS, new medium was added with resazurin at a final concentration of 88 μM. A stock solution of resazurin was prepared by filtering through Minisart® syringe filters with a pore size of 0.2 μm (Sartorius, Göttingen, Germany) and stored at 4°C for no longer than 1 week. Supernatant aliquots of 100 μl were transferred to a flat bottom Cellstar® plate (Greiner bio-one, Frickenhausen, Germany) at indicated time intervals and absorbance was measured at 600 nm on a Tecan Infinite M200 Pro plate reader (Tecan, Männedorf, Switzerland). Data displayed represent the change in resazurin absorbance relative to controls with no cells and unreduced resazurin.

### Microscopy imaging of biofilms

Biofilms for microscopic observation were generated for 24 h on ibiTreat plastic 35 mm high μ-dishes (Ibidi GmbH, Munich, Germany) with an initial inoculum density of 10^4^ spores in a total volume of 1.0 ml in RPMI supplemented with inducers as mentioned above. After 24 h of cultivation, biofilms were washed once with sterile PBS and stained for all polysaccharides with 1.0 ml of 25 μg/ml Concanavalin A, Alexa Fluor® 488 Conjugate (CAAF, Life Technologies, Darmstadt, Germany) for 45 min at 37°C in static conditions. Unbound CAAF was removed by three gentle washing steps and 1 ml of fresh PBS was added. Chitin structures were stained by adding 2.0 μl of 10 mg/ml of Fluorescent Brightener 28 (Sigma-Aldrich) for further 10 min at room temperature. Where indicated, samples were also labeled by addition of 5.0 μl of 1 mg/ml of the nucleic acid intercalating dye propidium iodide (PI, Sigma-Aldrich). This dye is membrane impermeable and stains eDNA and damaged or dead hyphae. Without further incubation biofilms were analyzed by inverse fluorescence microscopy using an Axiovert 200 M/LSM 5 live confocal laser scanning microscope (CLSM, Carl Zeiss, Jena, Germany). Fluorescence signals were detected using band pass filters of 415–480 nm (Fluorescent Brightener 28, Life Technologies), 560–675 nm (PI) and 500–525 nm (CAAF). All images were captured and processed using the ZEN 2008 software (Carl Zeiss, Jena, Germany).

### Statistics and data presentation

All data presented represent the means and standard deviations of at least three biological replicates. Graphical presentation and statistical evaluation of the experimental data using the unpaired Students' test (*t*-test) was carried out with Microsoft Office Excel 2007 software (Microsoft, Redmond, USA). *P*-values of <0.05 were considered as criteria for statistical significance.

## Results

### Extracellular DNA promotes biofilm growth of *A. fumigatus*

**Figure 1:**
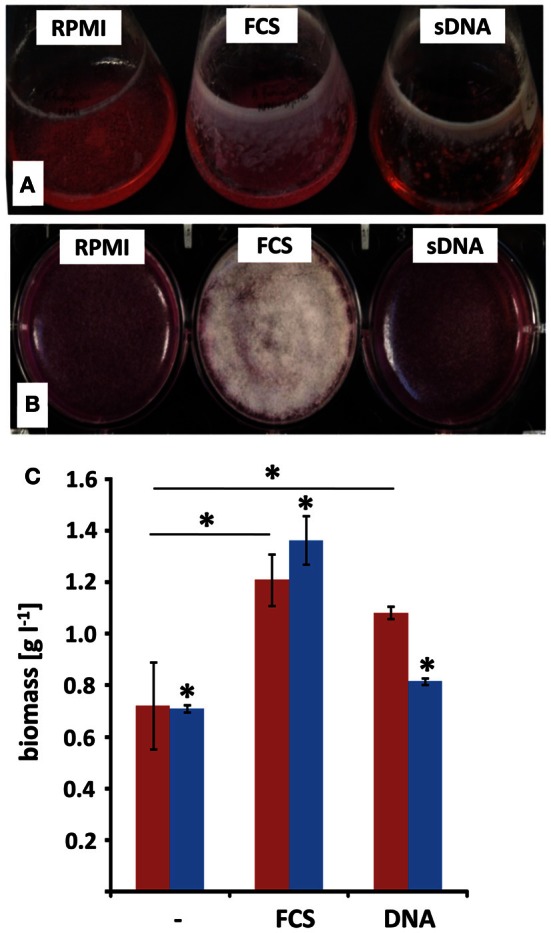
**Surface adhesion and biofilm formation with extrinsic DNA.** *A. fumigatus* conidia were grown either in RPMI without additives (RPMI), with addition of 10% [v/v] fetal calf serum (FCS) or with 1 mg ml^−1^ sheared herring sperm DNA (sDNA) in submerged, shaken culture **(A)** or in static biofilm inducing conditions **(B)**. Growth was monitored after 48 h and expressed as total biomass formation during shaking (blue) and static (red) culture conditions **(C)**. Displayed values represent the means and SDs of three independent experiments and asterisks indicate *p* < 0.05 in Student's *t*-test.

**Figure 2:**
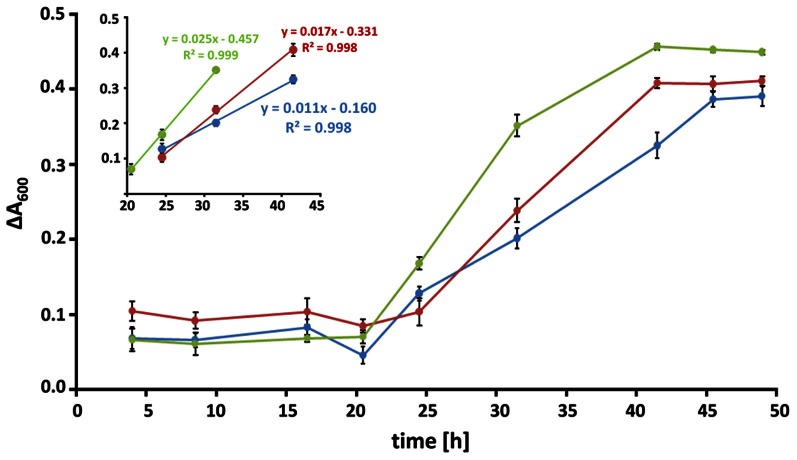
**Kinetics of biofilm formation.** Biofilms at conidial densities of 10^5^ per well were grown in RPMI alone (blue), in the presence of 10% [v/v] FCS (red) or 1 mg/ml of DNA (green) and fungal viability was monitored over time. Metabolic reduction of resazurin was measured as the decrease in absorbance at a wavelength of 600 nm. Data are expressed as the change in absorbance relative to an untreated control and represent the means and SDs of a biological triplicate. Time periods of linear growth kinetics for all three conditions are displayed in the inset.

RPMI medium contains all nutrients necessary for the growth of *A. fumigatus*. However, previous work showed that serum additions like 10% (v/v) FCS, led to the formation of adhesive biofilms consisting of a dense hyphal network and high amounts of ECM (Seidler et al., 2008; Bruns et al., 2010b). As eDNA was shown to be an abundant molecule in the lung tissues of patients suffering from pulmonary infections, we analyzed how eDNA affects biofilm growth *in vitro*. *A. fumigatus* biofilms have been reported to show a specific increase in biofilm biomass in comparison to biomass generated under shaking, planktonic conditions (Seidler et al., 2008). Hence, we determined the dry weight formation during these two growth conditions (Figures 1A,B). In RPMI alone biomass formation was nearly identical when the fungus grew in a planktonic state or as a biofilm. As expected, addition of FCS or eDNA led to increased biomass after 48 h in both growth forms when compared to RPMI medium alone (Figure 1C). However, only in the presence of eDNA the fungal biomass was specifically increased by at least 20% when *A. fumigatus* was cultured as a biofilm. Interestingly, similar biomass production in biofilms was seen for FCS and DNA, despite the macroscopic structures of the two biofilms varied drastically. While biofilms growing with DNA were more irregular and stayed entirely submerged leading to a gel-like appearance, FCS induced the formation of a surface mycelium which did not show any signs of conidia formation (Figure 1B). As a next step, we tracked the time-dependent increase in fungal viability in biofilms as a measure for growth (Figure 2). Fungal respiration due to enhanced metabolic activity results in the concomitant reduction of the redox indicative dye resazurin, which was determined as a decrease in absorbance. Initially conidia were seeded at a concentration of 10^5^ per well which was earlier found to be the optimal density for biofilm formation (Mowat et al., 2007). For all conditions metabolic activity was low during a lag period of 16–20 h, which was characterized by the conidial germination and the initial formation of hyphae. In the following 20 h the formation of mycelial monolayers was followed by rapid growth with nearly linear kinetics. In RPMI alone fungal cells reduced resazurin at a rate of 0.011 h^−1^, which rose to 0.017 h^−1^ with FCS (Figure 2, inset). Growth kinetics with eDNA were exceeding those obtained for FCS and were more than two fold higher when compared to RPMI alone, leading to a maximal reduction rate of 0.025 h^−1^. Similar kinetics were also reproducibly obtained when starting from a lower conidia inoculum of 10^4^ per well (not shown) further supporting the hypothesis that extrinsic DNA acts as an efficient inducer of biofilm growth.

### Extrinsic DNA induces conidial surface adhesion

**Figure 3:**
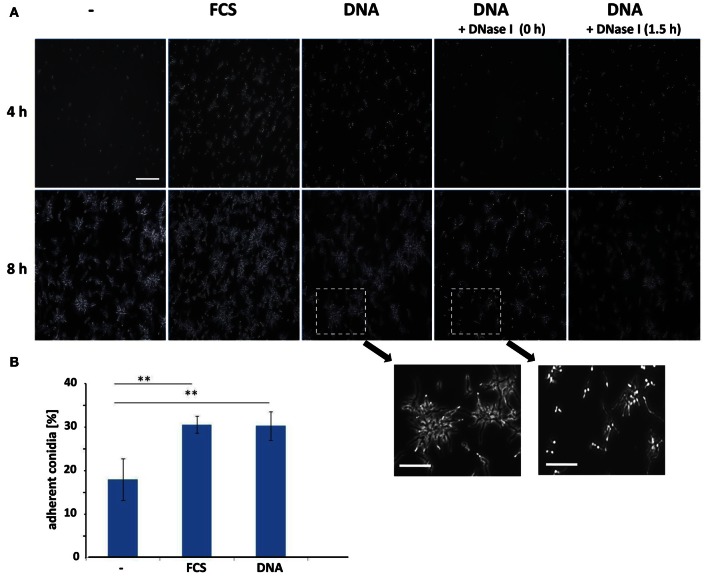
**Conidial surface adhesion is enhanced by extrinsic DNA. (A)** Conidia at an initial concentration of 2 × 10^6^ per well in RPMI were allowed to adhere to polystyrene surfaces in the presence of FCS or sheared DNA (sDNA) as additives. DNase I was added at indicated time points during the 4 h adhesion phase. After washing microscopy images were either taken directly following the adhesion phase (4 h, upper panel) or after germination (8 h, lower panel). Two enlarged regions illustrate the effect of DNase I treatment on conidial germination. The white bars indicate 100 μm or 50 μm (enlarged). Adherence of conidia was quantified by direct microscopic counting **(B)**. Data and SDs were based on four biological replicates and two asterisks indicate *p* < 0.01 in Student's *t*-test.

For bacteria, and especially for the opportunistic pathogen *P. aeruginosa*, it has been well-established that eDNA promotes biofilm formation already at the earliest stage of development (Whitchurch et al., 2002). Therefore, we analyzed whether the presence of DNA also influenced the initial phases of biofilm formation in *A. fumigatus*. For single cell organisms like bacteria, cellular adhesion is often quantified by counting the CFUs. This standard test proved to be difficult and produced unreliable data sets, presumably due to the extensive aggregation of fungal conidia often observed in liquid (Fontaine et al., 2010). As a consequence, we quantified surface adherent conidia directly by microscopic counting following a 4 h initial adherence phase (Figures 3A,B). This experimental approach revealed significant differences in conidial adhesion depending on the presence of FCS or DNA. The addition of any of the two components also did not alter germination, as imaging after 8 h indicated that both rate and frequency stayed essentially constant under all three conditions. In RPMI alone, after 4 h only few conidia had adhered to the surface of the well, but the presence of FCS or DNA increased adhesion by at least a factor of two. Addition of DNase I revealed that this process was indeed specific to DNA, as the treatment at early time points drastically reduced the ability of the conidia for surface adhesion down to RPMI comparable levels.

### Extrinsic DNA leads to production of matrix polysaccharides

**Figure 4:**
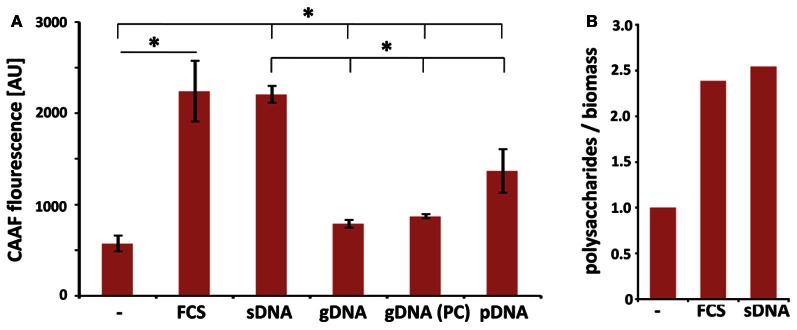
**Extrinsic DNA increases ECM formation in biofilms. (A)** CAAF fluorescence of total fungal polysaccharides was determined in biofilms seeded at an initial density of 10^4^ per well and used as a measure for ECM production in biofilms in the presence of 1 mg ml^−1^ DNA from indicated sources (sDNA, sheared herring sperm DNA; gDNA, genomic DNA from *A. fumigatus*; PC, phenol chloroform extracted DNA; pDNA; purified plasmid DNA). Displayed values represent the means and SDs of three independent experiments and an asterisks indicate *p* < 0.05 in an unpaired *t*-test. **(B)** FCS- and DNA-specific increases in total polysaccharide production are expressed as the ratio of CAAF fluorescence to biomass normalized to untreated controls.

Surface adhesive growth of *A. fumigatus* was promoted by the presence of DNA and dry weight measurements demonstrated an increased biomass production under these conditions. As a follow-up of this study we addressed the question whether increased viability also corresponds to enhanced formation of cellular polysaccharides. Concanavalin A coupled to fluorescent Alexa-fluor 488 (CAAF) specifically stains polysaccharides of the ECM and the fungal cell wall. Here, we also included DNA with different purities and from various sources and measured total fungal polysaccharides in mature biofilms after 48 h (Figure 4A). As expected, polysaccharide production with FCS or DNA was higher than in RPMI alone. When polysaccharide measurements were normalized to biomass, these values indicated that matrix formation was specifically enhanced by more than a factor of two in the presence of FCS and DNA (Figure 4B). DNA from different sources all enhanced polysaccharide production to different extent. Interestingly, ECM formation was also dependent on the length of the DNA fragments. The two DNA preparations with shorter DNA fragments (3.0 kbp for plasmid and ~0.05 kbp for sheared DNA) led to higher induction when compared to crude isolations of fungal genomic DNA (>30 kbp). Purity of the DNA was a minor factor as multiple phenol chloroform extractions of the genomic DNA had no significant influence on ECM production (Figure 4A).

### Structure of biofilms formed in the presence of eDNA

**Figure 5:**
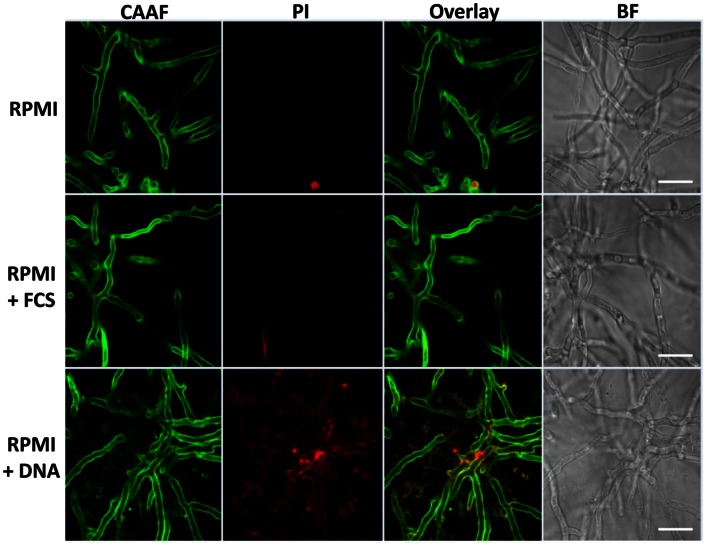
**Extrinsic DNA localizes to cellular polysaccharides.** Biofilms were grown for 24 h in RPMI with 10% [v/v] FCS and 1 mg ml^−1^ sheared DNA and stained for total polysaccharides using Concavalin A coupled to fluorescent Alexa-fluor 488 (CAAF) or for nucleic acid using propidium iodide (PI). The two right panels represent an overlay of CLSM images from the CAAF and PI channel and the according brightfield images (BF), respectively. White bars indicate 20 μm.

Biofilm structures of fungi can vary greatly with regard to surfaces, medium composition or even conidial seeding density. In *A. fumigatus* such variations are at least partially reflected by differences in the production of ECM. Hyphae found in aspergilloma are covered by an adhesive, polysaccharide-based ECM, providing structure and stability to the hyphal network (Loussert et al., 2010). Similar observations were made earlier for aerially grown colonies of the fungus or hyphae growing submerged under biofilm-inducing conditions (Beauvais et al., 2007; Mowat et al., 2007; Seidler et al., 2008). As eDNA revealed to be an efficient inducer of biofilm, we analyzed whether this also affected the structure of these biofilms. As mature biofilms were characterized by a massive formation of hyphal structures, leading to a thick network, which prevented the visualization of detailed structures, we focused on the end of the initial maturation phase after 24 h. At this time point CLSM imaging could still reveal detailed structural information on the developing complex three-dimensional networks originating from a single germinating conidium (Figure 5). CAAF staining indicated that RPMI-grown fungal populations were composed of weakly attached hyphae lacking ECM polysaccharides, and thus, showed essentially no biofilm formation. In FCS, hyphae showed augmented branching with a diameter in the range of 3.5–4.5 μm, a size considerably larger in comparison with hyphae grown in RPMI alone (2.2–3.5 μm). Positive CAAF signals in FCS samples clearly showed that the distal hyphal tips were the predominant site of ECM localization and the exopolymeric material appeared as a cloudy “halo” around the tips of the hyphae. In agreement with earlier observations (Beauvais et al., 2007), the cohesive properties of the ECM under these conditions was also apparent, showing that these polysaccharides not only yield a protective cover, but also form sticky threads connecting the hyphal tips. The presence of DNA had only a minor influence on the thickness of the hyphae with average diameters ranging from 2.9 to 3.2 μm. In accordance with a smaller hyphal diameter seen in the presence of eDNA, relatively small amounts of the ECM were observed around the hyphae or at their tips. Instead, under these conditions the ECM was widely distributed between the hyphae forming a loose network of polysaccharides within the biofilm.

**Figure 6:**
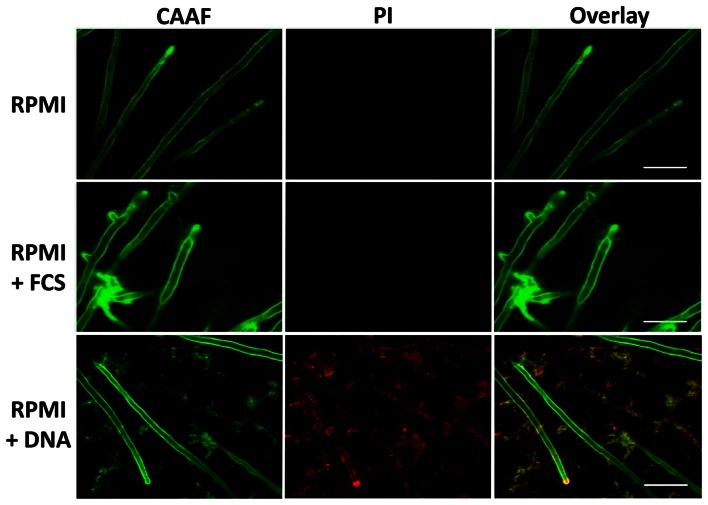
**Extrinsic DNA influences ECM distribution.** Peripheries of biofilms grown with RPMI with either FCS or sDNA were imaged by CLSM displaying the tips of terminal hyphae. Note the cohesive properties of ECM polysaccharides in the presence of FCS and the network-like distribution of the ECM in response to DNA. White bars indicate 20 μm.

Only in the presence of eDNA, the entire hyphal network appeared to be embedded in a diffusely localized ECM, similar as earlier described for mature biofilms (Seidler et al., 2008). This was even more pronounced when imaging the distal hyphae of the biofilms (Figure 6).

### Extrinsic DNA colocalizes with the extracellular matrix

**Figure 7:**
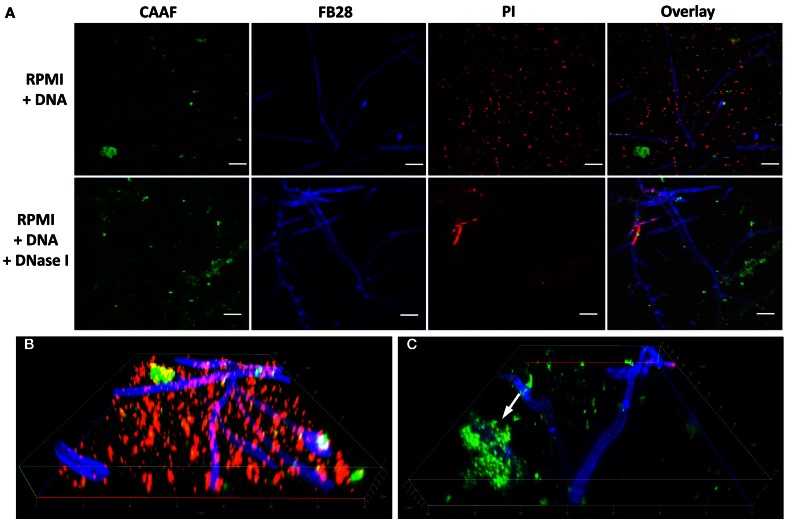
**Polysaccharides provide the main stability in early DNA induced biofilms.** Biofilms were grown with DNA over a time of 24 h and DNase I was added during early biofilm development (bottom panel). **(A)** Biofilms were imaged by CLSM after staining with CAAF for polysaccharides, with fluorescence brightener (FB28) for chitin and PI for DNA. White bars indicate 20 μm. Note that DNase I treatment completely diminishes the PI signal without changing the CAAF signal. Three dimensional reconstruction of DNA induced biofilms displaying the bedding layer network structure of polysaccharide and DNA before **(B)** and after DNase I treatment **(C)**. The white arrow indicates DNA entirely covered by polysaccharide and hence, presumably escaped enzymatic digestion.

In the polymeric matrices of a wide range of biofilms eDNA represents an important structural component (Martins et al., 2012; Rajendran et al., 2013). As we observed that DNA from extrinsic sources also induced and shaped biofilm formation, we addressed the question whether exogenous eDNA would also become incorporated into the ECM of *A. fumigatus*. Fluorescence staining of DNA with PI was previously shown to be a useful tool for DNA staining following membrane disruptions of bacteria, but also for the visualization of DNA structures and their localization during the formation of extracellular traps by human neutrophils. Young biofilms grown in the absence of eDNA showed only some residual staining when treated with this membrane impermeable, nucleic-acid-intercalating dye. Positive signals in DNA-free biofilms were restricted only to membrane disrupted fungal cells, as is expected for the origin of conidial germination and for a few dead hyphae (Figure 6). This finding further proved that PI was DNA specific and is in agreement with a recent study, showing that only mature biofilms release significant amounts of genomic DNA by autolysis (Rajendran et al., 2013). When biofilms were formed in the presence of eDNA, PI showed an identical signal distribution in the biofilm as seen for the extracellular polysaccharide-specific CAAF signal (Figure 6). To check whether these signals were specific and the DNA did indeed colocalize with the ECM, we used DNA of various lengths and sources. Only when DNA was subjected to DNase I digestion, PI staining of the biofilm was completely diminished (Figure 7). However, DNase I treatment did not promote any obvious structural changes at this stage of biofilm developement, as the overall three-dimensional distribution of polysaccharide in the hyphal network was not altered (Figures 7B,C). Furthermore, DNA bound to polysaccharide was mainly observed as a bedding layer of the biofilm, providing additional evidence for its functional role during the first stages of development.

## Discussion

Only in the past years increasing evidence has favored the idea that the hyphal network of filamentous fungi resembles biofilm structures. Up to now, however, there is only little knowledge about how filamentous growth contributes to the virulence of *A. fumigatus* during the different disease patterns caused by this opportunistic pathogen. During CF *A. fumigatus* is a common lung colonizer most likely favored by chronic antibiotic therapy in older patients (Pihet et al., 2009). The mucus-rich environment is characterized by high concentrations of free DNA originating from a subpopulation of attracted neutrophils undergoing NETosis. Using an *in vitro* model for biofilm development, we showed that eDNA supports the surface adhesion of fungal spores, and that the free DNA becomes part of the ECM surrounding the fungal biofilm cells.

Filamentous fungi such as *A. fumigatus* grow well under submerged shaking conditions in minimal medium, but first investigations demonstrated that addition of complex nutrient sources like FCS supports surface attachment and subsequent biofilm formation. Fetuin A was identified as one crucial serum component involved in this process (Toyotome et al., 2012), raising the question to which extent biofilm formation is favored by specific components present in FCS or complex nutritional environments. When we quantified total fungal dry mass produced under the different growth conditions, the nutrient-rich FCS resulted in an increase in biomass as expected. However, a biofilm-specific increase in dry weight was only seen upon addition of exogenous DNA, leading to the conclusion that this additive supported biomass formation primarily during static growth conditions. Enhanced biomass formation in the presence of FCS and DNA was also reflected by the increased viability in biofilms, measured as an increased reduction of resazurin to resofurin. It seemed likely that this specific increase in viability would also result in higher production of polysaccharides as part of the ECM, as also observed in prior studies on *A. fumigatus* biofilms (Seidler et al., 2008). For a detailed analysis of polysaccharide production during biofilm formation we employed a semi-quantitative assay based on CAAF fluorescence measurements in microtiter plates. Similar amounts of extracellular polysaccharides were produced when FCS was substituted by DNA, and correlated to the cellular viability under the same conditions. Also, when normalized to the biomass production, it became obvious that polysaccharide production was specifically induced in the fungus. Interestingly, polysaccharide production seemed to correlate negatively to the lengths of the DNA fragments, suggesting rather an electrostatic interaction than any type of structural recognition. For a further support we studied closer the initial phase of biofilm formation. Conidial surface adhesion was supported by eDNA to almost the same extent as FCS. It is interesting to note that nearly all adhered spores revealed synchronized germination, as judged by light microscopy during the initial phases of biofilm development following the 4h adherence phase. DNase I treatments throughout the adherence phase reduced the number of adherent spores, indicating the importance of DNA during adhesion. This is in perfect agreement with a recent study showing that DNase I treatment drastically disturbed the structural integrity of mature biofilms of *A. fumigatus* (Rajendran et al., 2013).

As a next step we studied whether eDNA had any influence on the structure and organization of the hyphal network. CLSM revealed that with FCS the ECM polysaccharides were primarily localized at the hyphal tips, thereby specifically enclosing individual hyphae during linear growth and connecting distant hyphae via cohesive threads. Previous work showed that biofilms comprise a dense hyphal network embedded in a cohesive polysaccharide matrix and similar structures were also observed for hyphae recovered from the lungs of aspergillosis patients, and even more pronounced for hyphae during aspergilloma (Beauvais et al., 2007; Seidler et al., 2008; Loussert et al., 2010). In the presence of DNA a smaller diameter of the hyphae was accompanied by a wide spread ECM that was shaped like a network, resembling the situation during static aerial colony formation (Beauvais et al., 2007). DNA colocalized with the polysaccharide network as revealed by the specific DNase I sensitive PI staining of the ECM. The electrostatic affinity of nucleic acids for polysaccharides of *A. fumigatus* was addressed previously, when hyphae were found to efficiently scavenge small RNAs and DNA oligonucleotides within their cell wall (Jöchl et al., 2009). It is therefore conceivable that the presence of free DNA might trigger enhanced polysaccharide synthesis, resulting in increased adhesion and ECM formation. Furthermore, DNA as a part of the ECM was primarily detected on the bottom, bedding layer of the biofilms, similar as seen earlier for young biofilms of *P. aeruginosa*, where eDNA formed grid-like structures on the substratum (Allesen-Holm et al., 2006). Also, for a number of other bacteria, DNA was shown to be a crucial component of the biofilm (see Flemming and Wingender, 2010 for review). In two recent studies this was also demonstrated for the fungal species *C. albicans* and *A. fumigatus* (Martins et al., 2012; Rajendran et al., 2013). For the latter one, structural support by autolysis-derived DNA is of major importance during later stages of biofilm development and was also proposed to be of medical importance due to reduced susceptibility to antifungals, which was partially counteracted by the concomitant addition of DNase (Rajendran et al., 2013). In agreement with this finding, we found that extrinsic DNA enhanced surface adhesion of conidia and shaped the organization and distribution of the ECM during early biofilm development. It will be interesting to analyze whether eDNA originating from neutrophils, serves as a crucial nutrient source for the fungus and contributes to biofilm formation *in vivo*, especially in the DNA-rich mucus environment in the lungs of CF patients.

### Conflict of interest statement

The authors declare that the research was conducted in the absence of any commercial or financial relationships that could be construed as a potential conflict of interest.
